# Pleomorphic Adenoma of the Nasal Vestibule: An Atypical Presentation

**DOI:** 10.22038/IJORL.2024.58890.3046

**Published:** 2024-03

**Authors:** Lady Johana Morales, Alejandro Uribe Escobar, Cesar Augusto Garcia

**Affiliations:** 1 *Department of Otolaryngology, Universidad de Cartagena.* * Calle 29 N C2 BA 50-50, ESE Hospital Universitario del Caribe, Cartagena, Bolivar, Colombia.*

**Keywords:** Benign mass, Head and neck mass, Salivary glands

## Abstract

**Introduction:**

We introduce the third case reported in the literature of an atypical presentation of pleomorphic adenoma located in the nasal vestibule of a young patient who assisted at our clinic.

**Case Report:**

A young man with no important medical history consulted due to a painless mass-type slow-growth lesion associated with right nasal obstruction. He underwent surgical management and complete resection of the mass. The pathological study revealed a pleomorphic adenoma, confirmed by immunohistochemistry.

**Conclusion:**

This case confirms that pleomorphic adenomas can occur anywhere in the head and neck, even in areas without upper air-digestive tract mucosa.

## Introduction

Pleomorphic adenomas, also known as mixed benign tumors, are characterized by the presence of mesenchymal and epithelial components in different proportions within the tumor, which dinstiguishes its pleomorphism and denomination as a mixed tumor. They represent 75% of benign tumors of the major salivary glands, occurring in a smaller proportion in the minor salivary glands (1).

## Case Report

A 25-year-old man, with no previous history of known diseases, consulted at the ENT service with approximately 5 months of complaint consisting of the appearance of a slow-growing mass at the level of the right nasal vestibule, approximately 1.5 x 1 x 1 cm, associated with right nasal obstruction. He denied pain, rhinorrhea, bleeding, or any other symptomatology.

**Fig 1:**
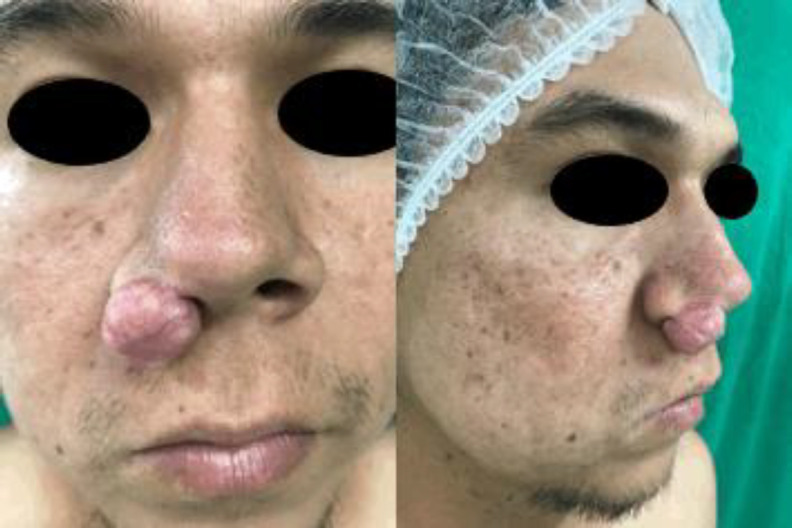
A. Front view of lesion in the right nasal ala, before the surgical procedure. B Lateral view of the same lesion

The mass had a rounded contour and a rubbery consistency, with a pedicle base to the right lateral nasal vestibule. Its outermost portion involved the skin of the alar ridge due to its size, which allowed it to oscillate, a characteristic that the patient took advantage of by introducing it endo-nasally as a way to hide the mass. In addition, the lesion had the presence of ectatic vessels on its surface, which were smooth. (Figure 1).

**Fig 2:**
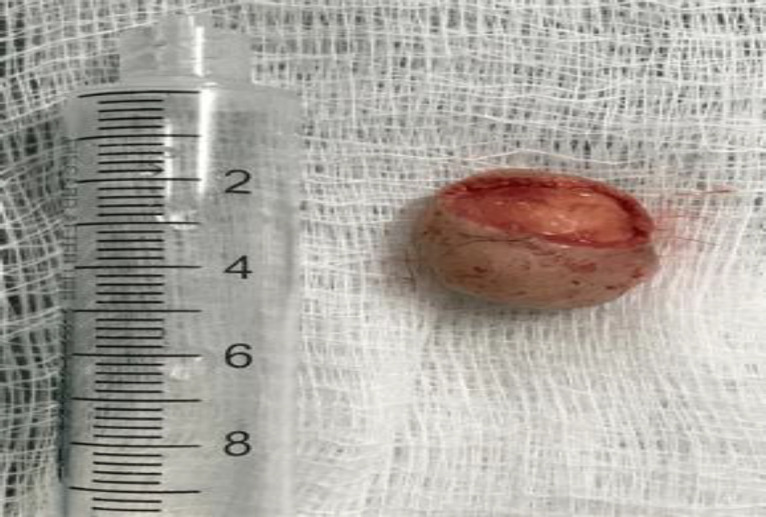
Image of the tumor after resection

The patient was taken to surgery, and complete resection of the lesion was performed, removing a small segment of skin from the vestibular area without exposing the alar cartilage and without compromising nasal support or aesthetics (Figure 2). 

**Fig 3:**
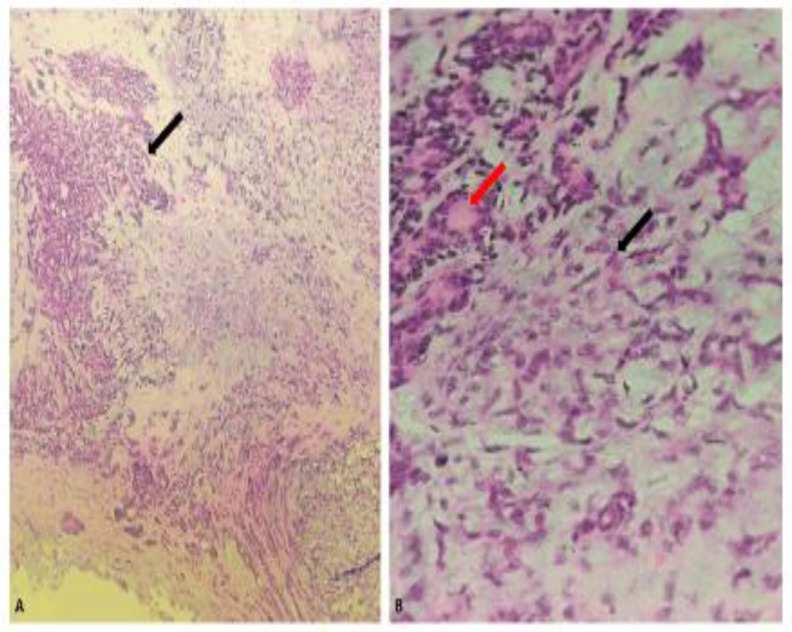
A. The proliferation of epithelial and myoepithelial cells (black arrow) immersed in a chondromyxoid stroma is observed. Hematoxylin and Eosin, 10x. B. Spindle-shaped myoepithelial cells (black arrow) embedded in the chondromyxoid stroma are recognized. Myoepithelial cells can release basement membrane-like material (red arrow) composed of laminin and type IV collagen. Hematoxylin and Eosin, 40x

The resected tissue was consequently sent for histopathological study. The postsurgical defect was small, so a secondary intention closure was considered. The histological specimen reported a completely resected benign neoplastic lesion with epithelial areas, formation of ducts and glandular structures (mixed) lined by cuboidal cells, foci of squamous cells interspersed with myxoid stroma, and foci of a chondroid appearance compatible with pleomorphic adenoma (Figure 3).

Given the atypical location of the lesion, an immunohistochemical study was requested to confirm the diagnosis. The studied cells were reported to be positive for cytokeratins and S100 diffusely and intensely, confirming the diagnosis.

## Discussion

Tumors affecting the salivary glands account for 3% of all head and neck tumors (1). Of these, 85 to 90% originate from the major salivary glands, where the pleomorphic adenoma is the most frequent and is commonly located in the parotid gland. Within the minor salivary glands, this type of tumor is infrequent and constitutes only 8% of cases, regularly located in the nasal septum (2). They can also occur anywhere minor salivary glands are found, such as the sinonasal cavities, external nose, neck, pinna, and mediastinum. Pleomorphic adenomas may appear on the skin due to ectopic salivary glands (3).

We present the third case of pleomorphic adenoma of the nasal vestibule reported in global literature. In both previous cases, the histopathology was similar to that reported in the usual locations (4, 5), and the treatment was the same: complete surgical excision to prevent recurrences. In the case reported by Baek et al. (4), there is limited clinical information about the mass, but the brief description included is very similar to the one we present. Along with its macroscopic aspect and its location in the nasal vestibule, the presence of ectatic blood vessels was also noted. In the other case reported by Hardeep et al. (5), the mass was more lateral to the right nasal vestibule without affecting nasal ventilation. The patient was more concerned about the aesthetic issue than the obstruction caused by the adenoma. A complete resection was completed in both cases, as in our case. Our patient has not presented recurrency 18 months after surgical treatment.

## Conclusion

Pleomorphic adenomas are rare benign tumors that can occur anywhere the minor salivary glands are located, including the external nasal region and the sinonasal cavities. 

The case presented, a pleomorphic adenoma of the right lateral nasal vestibule, represents an atypical presentation of these tumors and one of the few reported to date in the global literature. In this case, we confirmed that pleomorphic adenomas can occur in any part of the head and neck, even in those areas without mucosa of the upper digestive tract.

## References

[1] Saade RE, Bell DM, Hanna EY, Flint Paul W ( 2015). Benign Neoplasms of the Salivary Glands. Cummings Otolaryngology.

[2] Prager DA, Weiss MH, Buchalter WL, Jacobs M (1991). Pleomorphic adenoma of the nasal cavity. Ann Otol Rhinol Laryngol..

[3] Nishimura S, Murofushi T, Sugasawa M (1999). Pleomorphic adenoma of the auricle. European Archives of Oto-Rhino-Laryngology,.

[4] Baek BJ, Lee JY, Bae CH, Kang EG, Cho HD (2009). Clinical photographs Pleomorphic adenoma of the nasal vestibule. Otolaryngol Head Neck Surg..

[5] Irfan I, Kanwaljeet S, Hardeep S (2014). Pleomorphic Adenoma of the Nose. Indian Journal of Clinical Practice,.

